# Case Report: Interferon-γ Restores Monocytic Human Leukocyte Antigen Receptor (mHLA-DR) in Severe COVID-19 With Acquired Immunosuppression Syndrome

**DOI:** 10.3389/fimmu.2021.645124

**Published:** 2021-04-07

**Authors:** Steffen Dickel, Clemens Grimm, Katharina Amschler, Sebastian Uwe Schnitzler, Julie Schanz, Onnen Moerer, Didier Payen, Bjoern Tampe, Martin Sebastian Winkler

**Affiliations:** ^1^Department of Anesthesiology and Intensive Care Medicine, University Medical, Center Goettingen, Goettingen, Germany; ^2^Department of Dermatology, University Medical Center Goettingen, Goettingen, Germany; ^3^Institute of Clinical Chemistry and Laboratory Medicine, University Medical Center, Goettingen, Goettingen, Germany; ^4^Universite Paris 7 Cite Sorbonne, UMR INSERM 1160, Paris, France; ^5^Department of Nephrology and Rheumatology, University Medical Center, Goettingen, Goettingen, Germany

**Keywords:** SARS-CoV-2, COVID-19, monocytic human leukocyte antigen receptor, interferon-y, intensive care, acute respiratory distress syndrome, acquired immunosuppression syndrome, case report

## Abstract

**Background:**

The major histocompatibility complex (MHC) class II characterized by monocytes CD14+ expression of human leukocyte antigen receptors (HLA-DR), is essential for the synapse between innate and adaptive immune response in infectious disease. Its reduced expression is associated with a high risk of secondary infections in septic patients and can be safely corrected by Interferon-y (IFNy) injection. Coronavirus disease (COVID-19) induces an alteration of Interferon (IFN) genes expression potentially responsible for the observed low HLA-DR expression in circulating monocytes (mHLA-DR).

**Methods:**

We report a case of one-time INFy injection (100 mcg s.c.) in a superinfected 61-year-old man with COVID-19–associated acute respiratory distress syndrome (ARDS), with monitoring of mHLA-DR expression and clinical tolerance.

**Observations:**

Low mHLA-DR pretreatment expression (26.7%) was observed. IFNy therapy leading to a rapid increase in mHLA-DR expression (83.1%).

**Conclusions:**

Severe ARDS in a COVID-19 patient has a deep reduction in mHLA-DR expression concomitantly with secondary infections. The unique IFNy injection was safe and led to a sharp increase in the expression of mHLA-DR. Based on immune and infection monitoring, more cases of severe COVID-19 patients with low mHLA-DR should be treated by IFNy to test the clinical effectiveness.

## Introduction

Among the reported clinical phenotypes after severe acute respiratory syndrome coronavirus-2 (SARS-CoV-2) infection, acute respiratory distress syndrome (ARDS) is the most frequent life-threatening context requiring treatment in intensive care unit (ICU) (1). The underlying molecular mechanisms of the SARS-CoV-2 induced host response remain partially unknown. However, it is clear that inflammation patterns can rapidly change over time (2, 3). We have recently shown that an early and sharp decrease in monocytic human leukocyte antigen receptors (HLA-DR) expression in peripheral blood was associated with changes in the proportion of monocyte subpopulations (2). Since the impairment of interferon (IFN) gene expression have been demonstrated, a deficit in interferons has been suspected with a potential treatment option with IFN molecules to reinforce immune defenses (4). This is the first case report of a patient with severe ARDS from COVID-19 suffering from multiple secondary infections, associated with a dramatic reduction in mHLA-DR expression on circulating monocytes. Even after several days of adequate pharmacologic antimicrobial treatment the patient displayed ongoing clinical deterioration, repetitive positive blood cultures and low expression of mHLA-DR. This led to the compassionate use of one dose of IFNy to boost innate immunity and antigen presentation.

## Medical History

A 61-year-old man presented himself to his family physician two days after the onset of an irritable and dry cough. Preexisting medical conditions included cardiovascular risk factors, arterial hypertension, nicotine abuse and obesity. SARS-CoV-2 RNA was confirmed on day three after onset of symptoms. On this day he was admitted to a regional hospital ICU due to a continuous decline of his physical status. Severe hypoxia was first treated by non-invasive, then invasive ventilation and subsequently by insertion of veno-venous extracorporeal membrane oxygenation (ECMO) on the twelfth day of the illness. The patient was then transferred to our ARDS center with the diagnosis of superinfected COVID-19 ARDS.

## Clinical Course

**Figure 1:**
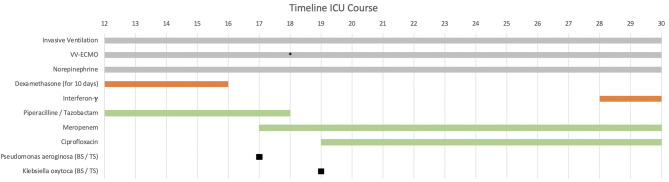
Patient’s clinical history from admission to the university center intensive care unit (ICU) on day 12 after first symptoms. The immunomodulatory therapy is shown in red and the antibiotic therapy in green. Additional ECMO cannulation on day 18 (marked with *), dexamethasone administration 6 mg intravenously (i.v.) per day for 10 days (started before ICU admission). Interferon-y (IFNy) administration on the 28th day with a single dose of 100 mcg subcutaneously (s.c.). vv-ECMO (veno-venous extracorporeal membrane oxygenation). BS/TS (found in blood culture and tracheal secretion).

Immediately after ICU admission the patient’s condition worsened and tolerable blood gases (therapeutic goals: paO > 60 mm Hg, SpO > 90% and paCO 40–50 mm Hg) were only achieved under high-flow ECMO with 5.5 L/min blood- and 10 L/min of gas-flow (FiO 1.0). Several ventilation regimes were applied to optimize gas exchange under ECMO such as two-level ventilation with neuromuscular blockade and nitric oxide. To overcome this critical hypoxemia a secondary venous cannula was implanted on day 18 to increase ECMO blood-flow up to 7 L/min (**Figure 1**). The main clinical parameters are presented in **Table 1**. According to our standard operating procedure (SOP), the patient received intravenously (i.v.) dexamethasone for 10 days (6 mg per day). The diagnosis of a superinfected COVID-19 ARDS was made by the detection of Haemophilus influenzae, which was first treated with piperacillin/tazobactam for 6 days. Antibiotic treatment was switched to meropenem and ciprofloxacin after detection of a resistant Pseudomonas aeruginosa (3-MRGN) and a non-resistant Klebsiella oxytoca (**Figure 1**).

**Table 1:** Clinical findings and laboratory markers of the COVID-19 patient after ICU admission.

	After onset of first COVID-19 symptoms*									
	Normal range	Day 12*	Day 14	Day 16	Day 18	Day 20	Day 22	Day 25	Day 26	Day 29
Laboratory markers										
Leukocytes (×10^3^/μl)	4.0–11.0	15.40	17.50	16.30	11.87	13.10	10.60	8.54	6.99	7.10
CRP (mg/L)	≤5	316.8	351.3	432.4	324.0	N/D	313.9	242.8	239.4	190.7
PCT (μg/L)	<0.07	0.39	0.37	0.47	1.04	0.69	1.15	0.69	0.72	1.43
IL-6 (pg/ml)	<7.0	164.3	146.8	472.6	238.6	262.5	285.9	187.2	72.9	120.4
Ventilation										
Horowitz-Quotient	>300	127.5	97.8	116.9	56.0	65.0	69.0	80.0	57.0	74.6
*ECMO*										
Flow (1/min)		4.2	4.3	5.0	8.0	7.0	7.7	7.0	7.3	7.3
Clinical Finding										
SOFA-Score		10	11	11	12	16	15	15	16	15
Medication										
Norepinephrine in (μg/kg/min)		0.01	0.04	0.25	0.28	0.25	0.22	0.16	0.11	0.08

*Days after first COVID-19 symptoms, Admission to ICU (ARDS center) on day 12.

SOFA, sepsis-related organ failure assessment score; N/D, not determined.

## Diagnostics

**Figure 2:**
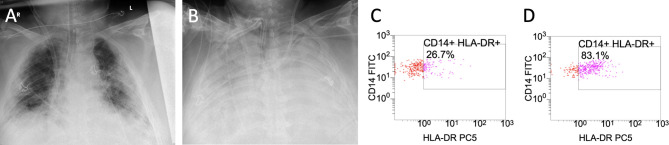
Caption shows the x-ray findings of the patient’s lungs on day 12 **(A)** and on day 28 **(B)**. Flow-cytometry (FACS) results of monocytic human leukocyte antigen receptors (mHLA-DR) on monocyte (CD 14+) before administration of interferon-y (IFNy) on day 26 **(C)** and after interferon-y (IFNy) treatment on day 29 **(D)**.

Sequential chest X-ray examinations showed infiltration, congestion and severe pneumonia (**Figure 2**). Routine inflammatory markers such as leukocytes, C-reactive protein (CRP), procalcitonin (PCT) and interleukin-6 (IL-6) are presented in **Table 1**. We extended our immune monitoring to flow-cytometry analysis (FACS; measured on a Navios^®^ system) of CD14+ monocytes and CD3+ lymphocytes, as shown in **Table 2**. Above all, we noticed a profound reduction in absolute number of monocytes that poorly expressed HLA-DR (CD14+ mHLA-DR+) within 22.4% to 28.4% on repetitive measurements, strongly suggesting a severe acquired immunodepression.

**Table 2:** Flow-cytometry (FACS) results.

Parameters	Day 16*	Day 18	Day 26	Day 29
Monocytes (cells/μl)	378	203	125	111
CD14 + (cells/μl)	325 (85.3%)	160 (78.9%)	97 (78.0%)	91 (82.0%)
CD14 + HLA-DR + (cells/μl)	73 (22.4%)	45 (28.4)	26 (26.3%)	75 (82.2%)
Lymphocytes (cells/μl)	1699	1210	1507	1181
CD3 + (cells/μl)	875 (51.5%)	734 (60.7)	1014 (67.3%)	858 (72.6%)
HLA-DR + (cells/μl)	126 (14.4%)	67 (9.1%)	87 (8.6%)	76 (8.9%)

*Days after first COVID-19 symptoms, admission to ICU (ARDS center) on day 12.

SOFA, sepsis-related organ failure assessment score), N/D not determined; CD, Cluster of differentiation, HLA-DR, human leukocyte antigen receptors.

## Intervention

The extreme severity, the repetitive secondary infection, the dramatic reduction in mHLA-DR expression and the stagnant clinical status after 16 days on our ICU prompted the off-label use of subcutaneously (s.c.) INFy in order to attenuate the acquired immunosuppression. We administered 100 mcg IFNy on day 28 after written consent by the patient’s legal representative. Monocytic HLA-DR expression levels were measured the day after.

## Outcomes/Results

After a single dose of IFNy the proportion of HLA-DR+/CD 14+ cells increased by 56.4% (**Table 2**). There was no impact on mHLA-DR expression in lymphocytes (CD 3+). Unfortunately, the patient died from refractory ARDS 29 days after the first symptoms of COVID-19.

## Discussion

The reported case of a patient with severe COVID-19 ARDS treated for 29 days in ICU matches the “typical” severe cases of COVID-19 pneumonia. The nonspecific inflammatory markers such as leukocytes or IL-6 are often moderately increased and do not necessarily correlate with the clinical severity, as observed in our case with low IL-6 levels and normal leukocytes (5, 6) (**Table 1**). The reported “hyperinflammation and cytokine storms” have also been discussed as a promoter of severe COVID-19 (7, 8), despite the big differences when compared to severe sepsis or septic shock (6). The consecutive overactivation of the adaptive immune system has been associated with lung failure and death (7–9). This background is one rationale for initial dexamethasone treatment, which had been also applied in our patient (10, 11). However, during the course of COVID-19 some patients develop signs of severe immunosuppression (8). This has been shown in longitudinal monitoring, when patients elicited markers of severe immunosuppression as observed in sepsis (2, 12, 13). Although not routinely incorporated in current sepsis care guidelines despite existence of functional cellular markers (13–15), the detection of an acquired immunosuppression syndrome (AIS) seems to be essential to tailor the immune therapy (16). Reduced mHLA-DR expression impairs the T-cells activation, which increases the likelihood of secondary infections by five times and is per-se associated with endotoxin tolerance (17, 18). Measurements beginning on day 17 of the illness revealed that merely 22% to 28% of CD14+ monocytes expressed HLA-DR. This supported our diagnosis of a severe AIS with high risk for secondary infection. The documented presence of opportunistic pathogens (Haemophilus influenzae, Pseudomonas aeruginosa and Klebsiella oxytoca) fits well with this concept. The timeline of our case corresponds to a recent longitudinal study of fifteen COVID-19 patients showing reduced mHLA-DR expression between days 11 and 14 (2). Taken together, these data support the hypothesis of a critical role of antigen presentation in COVID-19 and its potential impact on secondary infections and survival (2, 16, 19). In general, reduced HLA receptor status is an important risk factor for secondary infections in ICU (3, 19). Therefore mHLA-DR might present a comprehensive cellular and functional marker to detect the phase of highest vulnerability for secondary complications, as reported in COVID-19 (2). Interestingly, the bacterial superinfection occurred within this period in our patient. The unique dose of IFNy was able to restore mHLA-DR expression by 56.4%. To the best of our knowledge this is the first case report testing the efficacy and tolerance of IFNy therapy to overcome the COVID-19–induced AIS. Although this salvage therapy did not allow the patient to survive, it is important to note that circulating monocytes responded well after only one dose. IFNy treatment was justified by compassionate use and based on several case reports. IFNy treatment was done in close consultation with the patient’s relative and was concurrent with the patient’s probable will. The patient’s further clinical deterioration most likely did not result from the administration of IFNy. The safety of the drug has been demonstrated in various studies to date (20, 21). Known side effects include flu-like symptoms, fever, headaches, fatigue, diarrhea and rarely neutropenia and thrombocytopenia, which were not observed in this case after a single dose of IFNy (20, 21). Consequently, it is probably reasonable to suspect that a single dose was insufficient and the delay to treatment too long to reverse the disease. Our case report describes one patient with an acquired immunosuppressive syndrome potentially induced by SARS-CoV-2, which was first identified at day 12 after COVID-19 symptoms. Reduced mHLA-DR is a well-described and reliable marker for sepsis induced immune paralysis and a risk factor for secondary complications (19, 22). Our single observation is in line with reports that COVID-19 patients present significant reduced mHLA-DR levels between days 11 and 14 of their critical illness, when compared to earlier stages of the disease (2). We have learned, that for ICU supportive care phenotyping individuals and monitoring the immune status to identify a “period at risk” for secondary complications seems essential. Treatment of COVID-19 within this period with an old molecules such as IFNy might be one option to restore the COVID-19 sepsis–induced immune paralysis. Furthermore, immune monitoring is required since therapies, which have a broad impact on the immunity such as dexamethasone, are standard of care. Additionally, the consequences on other cells and cytokine levels will have to be investigated to ensure that IFNy does not impair tissue recovery or induce specific side effects such as fever or leukopenia (23, 24).

## Conclusion

Even though incompletely understood, the COVID-19 immune response moves along time evolution. After a few days, an AIS seems to be very frequent, which may facilitate the occurrence of secondary infections. This may prolong the ICU length of stay and may worsen the prognosis. Among the potential drugs shown to improve AIS, IFNy has specific targets on innate cells, with modest or no effect on adaptive immunity. Although more information is needed, it seems reasonable to monitor the mHLA-DR in association with microbiological screening in order to optimize the timely application and the duration of treatment with IFNy. Further studies will provide important information whether the application of IFNy should play an extended role in the safe and efficient treatment of AIS in COVID-19.

## Patient Consent

The case described does not contain any information on the identity of the patient.

## Data Availability Statement

The original contributions presented in the study are available from the corresponding author on reasonable request. Further inquiries can be directed to the corresponding author.

## Author Contributions

SD, CG, SS, OM, and MW treated the patient in ICU. SS and MW initiated the interferon therapy. JS performed the FACS analysis. SD, CG, DP, KA, BT, and MW analyzed the case and the laboratory findings. SD, CG, DP, and MW wrote the manuscript. All authors read and accepted the final version of the manuscript. All authors contributed to the article and approved the submitted version.

## Funding

The publication of the case report was supported by the open access publication fund of the Goettingen University Medical Center.

## Conflict of Interest

MW received unrestricted funding from SARTORIUS Ag-Lung research.

The remaining authors declare that the research was conducted in the absence of any commercial or financial relationships that could be construed as a potential conflict of interest.
